# Antibodies against Pneumococcal Capsular Polysaccharides and Natural Anti-Galactosyl (Alpha-Gal) in Patients with Humoral Immunodeficiencies

**DOI:** 10.1155/2017/7304658

**Published:** 2017-12-17

**Authors:** P. Kralickova, J. Kuhnova, O. Soucek, P. Vodarek, P. Zak, M. Simkovic, M. Motyckova, L. Smolej, E. Mala, C. Andrys, J. Krejsek, V. Thon

**Affiliations:** ^1^Institute of Clinical Immunology and Allergology, Faculty of Medicine in Hradec Kralove, Charles University and University Hospital Hradec Kralove, Hradec Kralove, Czech Republic; ^2^Department of Mathematics, Faculty of Science, University of Hradec Kralove, Hradec Kralove, Czech Republic; ^3^4th Department of Internal Medicine-Haematology, Faculty of Medicine, Charles University and University Hospital Hradec Kralove, Hradec Kralove, Czech Republic; ^4^Department of Clinical Immunology and Allergy, Faculty of Medicine, Masaryk University, St. Anne‘s University Hospital, Brno, Czech Republic; ^5^RECETOX, Faculty of Science, Masaryk University, Brno, Czech Republic

## Abstract

Humoral deficiencies represent a broad group of disorders. The aim of the study was to compare the levels of antibodies against pneumococcal capsular polysaccharides (anti-PCP) and natural anti-galactosyl (anti-Gal) antibodies in (1) patients with chronic lymphocytic leukaemia (CLL), (2) patients with common variable immunodeficiency (CVID), and (3) a healthy population and to explore their diagnostic and prognostic potential. Serum immunoglobulin levels and levels of anti-Gal IgG, IgA, and IgM and anti-PCP IgG and IgG2 were determined in 59 CLL patients, 30 CVID patients, and 67 healthy controls. Levels of IgG, IgA, IgM, anti-Gal IgA, anti-Gal IgM, and anti-PCP IgA were lower in CLL and CVID patients than in healthy controls (*p* value for all parameters < 0.0001). Decrease in the levels of IgA, IgM, anti-Gal IgA, and anti-PCP IgA was less pronounced in the CLL group than in the CVID group. IgA decline, anti-Gal IgA, anti-PCP IgA, and anti-PCP IgG2 were negatively correlated with CLL stage. We devise the evaluation of anti-Gal antibodies to be a routine test in humoral immunodeficiency diagnostics, even in cases of immunoglobulin substitution therapy. Significant reductions, mainly in anti-Gal IgA, IgM, and anti-PCP IgA levels, may have prognostic importance in CLL patients.

## 1. Introduction

B cell lymphocytic leukaemia (CLL) is the most common leukaemia in western European adults [1]. CLL is particularly frequent in the elderly population, with an average age at diagnosis of 72 years. The disease course and survival time are widely variable [1, 2]. CLL is characterized by an accumulation of clonal lymphocytes with a specific immunophenotype (CD5^+^, CD19^+^) in the bone marrow, peripheral blood, and secondary lymphoid organs and leads to organomegaly and suppression of physiological haematopoiesis [2]. Another clinical feature of CLL is complex alterations of the immune system, leading to higher susceptibility to infections, higher incidence of secondary malignancies, and autoimmune phenomena, such as autoimmune haemolytic anaemia and immune thrombocytopaenia [3, 4]. Infectious complications are the major cause of morbidity and mortality in more than 50% of all CCL-related deaths [5, 6]. The most common immune system defect in CLL patients is hypogammaglobulinaemia. Its severity correlates with the duration and stage of disease and is observed even in patients who have never been treated for CLL [7].

The main consequence of hypogammaglobulinaemia is increased frequency of respiratory tract infections caused by encapsulated bacteria (*Streptococcus pneumoniae*, *Haemophilus influenzae*, and *Staphylococcus aureus*) [8]. CLL patients with antibody failure must be identified based on immunization and monitoring, and patients who are clinically symptomatic should be protected by administration of prophylactic antibiotics and/or immunoglobulin substitution therapy [5, 9–11].

Common variable immunodeficiency (CVID) is a disease (or likely group of diseases) caused by a deficiency in primary antibody production with enormous heterogeneity in clinical presentation. The patients are characterised by decreased IgG levels, accompanied by decreased IgA and/or IgM levels, and highly disturbed specific antibody responses to antigen challenge. Other types of hypogammaglobulinaemia must be distinguished from this type [12]. Recurrent bacterial infections of the respiratory and gastrointestinal tracts are common symptoms. A significant proportion of patients also exhibit different features of immune dysregulation, including autoimmune diseases, lung granulomatous/interstitial inflammation, enteropathy, and malignancy [13, 14]. Adequate long-life immunoglobulin substitution therapy is indicated for all established CVID patients.

Evaluation of antibody production capacity is important in humoral immunodeficiency diagnostics and is a key criterion for the indication of immunoglobulin substitution therapy. Patients with hypogammaglobulinaemia should be examined for responses to T-dependent and T-independent antigens [11, 12]. Evaluation of natural antibodies may also be important.

Anti-galactosyl (anti-Gal) antibodies are the most abundant natural antibodies in humans and are naturally produced in apes and Old World monkeys. The ligand of anti-Gal is a carbohydrate antigen with the structure anti-Gal: *α*1-3Gal*β*1-4GlcNAc-R [15]. These antibodies can be detected in IgG, IgA, IgM, and IgE. Anti-Gal IgE is produced in some individuals and causes allergies to red meat, bovine gelatin, and cetuximab [16, 17]. Anti-Gal IgM and IgG also mediate rejection of xenografts expressing the *α*-gal epitope [15].

There is no detectable anti-Gal IgM and IgA in the cord blood of newborns, whereas anti-Gal IgG is present at similar levels in both the neonate and mother because of transplacental transfer during pregnancy. These antibodies are subsequently replaced by anti-Gal antibodies produced by the neonate, and their levels increase significantly during the first two years of life [18]. The plasma concentrations remain mostly stable, with some interindividual variability throughout the patient's life. Bernth-Jensen et al. found lower plasma levels of these antibodies in subjects with blood group B [19].

The aim of the study was to evaluate the diagnostic and prognostic role of antibodies against pneumococcal capsular polysaccharide (anti-PCP) and natural anti-Gal antibodies in CLL patients with secondary antibody deficiencies and patients with CVID.

## 2. Material and Methods

### 2.1. Study Design

The study protocol was approved by the local ethics committee. Participants in this study comprised a Czech population (Caucasian), including 59 patients with CLL (age range, 34–88 years, mean age, 66 ± 10 years; 38 men, 21 women), 30 CVID patients (age range, 18–82 years, mean age, 46 ± 16 years; 12 men, 18 women), and 67 sex- and age-matched healthy individuals (age range, 20–86 years, mean age, 56 ± 17 years; 37 men, 30 women).

The CLL patients did not receive any immunoglobulin replacement therapy or chemotherapy, anti-CD-20, or steroid therapy. Rai stage at the time of blood sample collection was 0, 1, 2, 3, and 4 in 14, 8, 14, 15, and 8 patients, respectively. Other characteristics of the CLL cohort are summarized in Table 1. All CVID patients fulfilled the criteria of the Pan-American Group for Immunodeficiency and European Society for Immunodeficiencies [20]. They were regularly administered intravenous (*n* = 21) or subcutaneous (*n* = 9) immunoglobulins at monthly doses of 368 ± 149 mg/kg (range, 170–940 mg/kg), with intervals between administrations individualized to maintain sufficient IgG trough levels. CVID patients were vaccinated during the diagnostic process for suspected immunodeficiency with the Pneumo 23 vaccine (Polysaccharidum Streptococci pneumoniae type: 1, 2, 3, 4, 5, 6B, 7F, 8, 9N, 9V, 10A, 11A, 12F, 14, 15B, 17F, 18C, 19A, 19F, 20, 22F, 23F, 33F, and 25 *μ*g for each serotype provided by Sanofi Pasteur, Lyon, France) between 2 and 10 years before inclusion in this study. They showed lower than 4-fold increases in anti-PCP IgG 3-4 weeks after immunisation. CLL patients and healthy controls were never vaccinated with any antipneumococcal vaccine.

Before intravenous immunoglobulin administration, blood samples were collected from patients by venipuncture, allowed to clot naturally, and then the serum was separated and stored at −80°C. Each sample was thawed once and tested for total IgG, IgA, and IgM; anti-Gal IgG, IgA, and IgM; and anti-PCP IgG and IgA in all groups. Anti-PCP IgG2 was only assessed in controls and the B-CLL group. The ratio of serum anti-Gal IgG, IgA, and IgM (U/mL) to the corresponding total serum IgG, IgA, or IgM (g/L) in U/mg and ratio of serum anti-PCP IgG and IgA (mg/L) to the corresponding total serum IgG, IgA, or IgM were calculated in mg/L.

### 2.2. Serum IgG, IgA, and IgM Levels

Serum levels of IgG, IgA, and IgM were determined by immunonephelometry (Immage 800, Beckman-Coulter, Brea, CA, USA). Data were obtained in g/L.

### 2.3. Serum Anti-Gal IgG, IgA, and IgM Levels

The serum concentration of anti-*α*Gal was determined by a sandwich enzyme-linked immunosorbent assay (ELISA), using an ELISA kit for human anti-alpha galactosyl IgG, IgA, and IgM (BioVendor, Laboratorní Medicína a.s., Brno, Czech Republic) according to the manufacturer's instructions. The range of detection was 3.13–100 U/mL for every kit. Serum samples were diluted 100x, and absorbance was read at 450 nm using a Multiskan RC ELISA reader (Thermo Fisher Scientific, Waltham, MA, USA).

### 2.4. Serum Anti-PCP IgG, IgG2, and IgA Levels

The serum levels of anti-PCP were determined by ELISA using specific VaccZyme™ ELISA kits for human anti-PCP IgG, IgG2, and IgA (Binding Site, Birmingham, UK), according to the manufacturer's instructions. The range of detection was 3.3–270 mg/L for the IgG kit, 1.1–90 mg/L for the IgG2 kit, and 0–270 U/mL for the IgA kit. Serum samples were diluted 100x, and the absorbance was read at 450 nm using a Multiskan RC ELISA reader (Thermo Fisher Scientific).

### 2.5. Data Analysis

Nonparametric tests were used to analyse the data because of insufficient numbers of observations and skewed readings. Spearman's rank correlation coefficient was used to determine the dependency of each parameter on age and, for CLL patients, disease stage. The Kruskal-Wallis test was used to compare all groups, followed by the Wilcoxon two-sample rank-sum test to compare each group by two when the Kruskal-Wallis test rejected the null hypothesis. All *p* values were adjusted using Bonferroni correction, and *p* < 0.05 was considered significant. Statistical analysis and other computations were performed using R language (Core Team, Vienna, Austria, 2016)

## 3. Results

**Figure 1:**
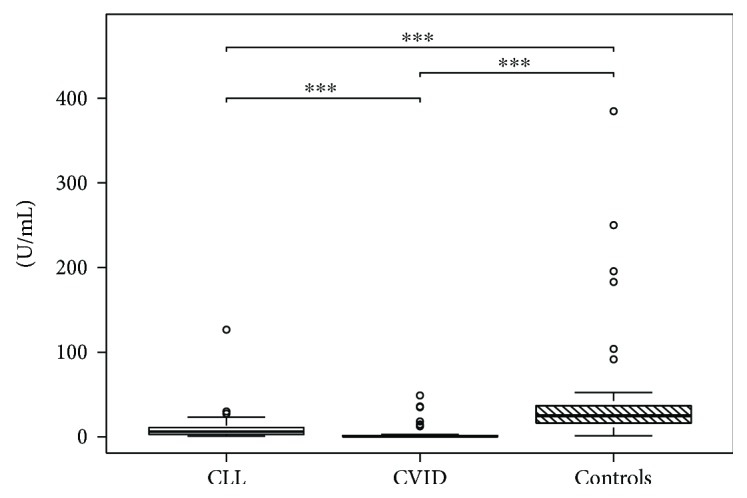
Comparison of anti-Gal IgA (U/mL) in peripheral blood. Results are presented as box plots with the median, first and third quartiles, and whiskers with 1.5 times the interquartile range marked with statistically significant pairs of groups. All groups showed significant differences (*p*^∗∗∗^ < 0.001) for anti-Gal IgA.

**Figure 2:**
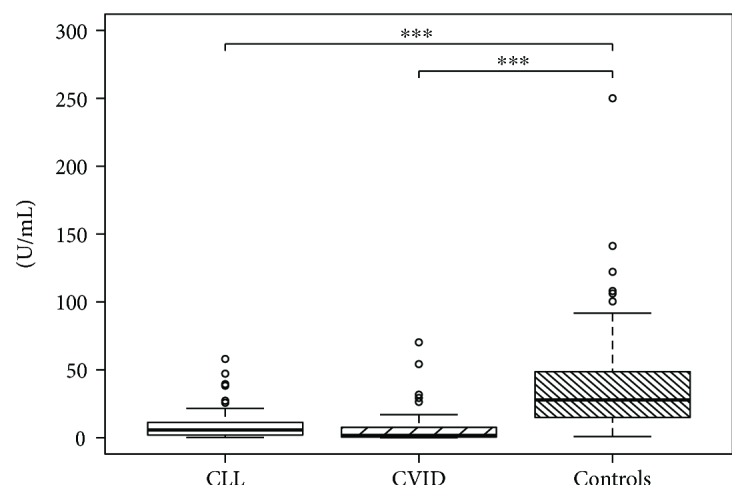
Comparison of anti-Gal IgM (U/mL) in peripheral blood. Results are presented as box plots with the median, first and third quartiles, and whiskers with 1.5 times the interquartile range marked with statistically significant pairs of groups. CLL and CVID groups showed significantly lower anti-Gal IgM level than healthy controls did (*p*^∗∗∗^ < 0.001).

**Figure 3:**
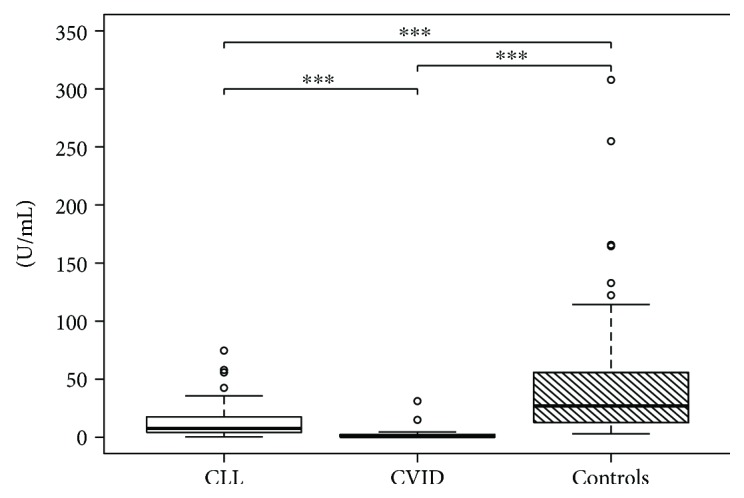
Comparison of anti-PCP IgA (mg/L) in peripheral blood. Results are presented as box plots with the median, first and third quartiles, and whiskers with 1.5 times the interquartile range marked with statistically significant pairs of groups. All groups showed significant differences (*p*^∗∗∗^ < 0.001) in anti-PCP IgA.

Descriptive statistical data for all parameters are shown in Tables 2 and 3. There was no statistically significant correlation with age for any parameters in our cohort (data not shown). Comparisons between groups are shown in Table 4 and Figures 1, 2, and 3 using box plots with the median, first and the third quartiles, and whiskers with 1.5 times the interquartile range marked with the statistically significant pairs.

In the CLL group, we observed a decrease below the normal range for age in at least one of the immunoglobulin classes in 38/59 (64.4%) CLL patients: IgG < 7.3 g/L in 18/59 (30.5%) patients; IgA < 0.8 g/L in 27/59 (45.7%) patients; and IgM < 0.4 g/L 28/59 (47.5%) patients. An IgG level less than 4 g/L was only observed in 4/59 (6.7%) patients. Although, anti-PCP IgG and anti-PCP IgG2 (CLL) did not differ significantly from controls, 15/59 (25.4%) CLL patients had anti-PCP IgG lower than 20 U/mL. The levels of IgA (*p* = 0.0118; *r* = −0.4306), anti-Gal IgA (*p* = 0.0286; *r* = −0.3982), anti-PCP IgA (*p* = 0.0085; *r* = −0.4374), and anti-PCP IgG2 (*p* = 0.0363; *r* = −0.3898) were negatively correlated with disease stage.

The ratio to the serum immunoglobulin level was measured. In CVID patients, the ratio of anti-PCP IgG (*p* < 0.00001) to serum IgG level was significantly higher than that in controls. In the CLL group, the ratios of anti-Gal IgA (*p* = 0.0004) and anti-Gal IgM (*p* = 0.0012) to total IgA/IgM were already lower than those in controls. No significant difference in the anti-Gal IgG to total IgG ratio in all groups and anti-PCP IgG2 and IgA to total IgG/IgA ratios between CLL patients and controls (data not shown). In CVID group, this comparison was not made for IgA and IgM because of the significant number of small values in most parameters in CVID patients.

## 4. Discussion

Hypogammaglobulinaemia is the most common immunodeficiency in CLL and occurs in more than 85% patients at some point during the disease [5] and in 25% of newly diagnosed patients [21]. Its prevalence and extent correlate with disease duration, advancing stage, and infection frequency [21–26]. Hypogammaglobulinaemia occurs in 10% of patients with Binet stage A [27] and up to 100% of patients with Binet stage C [28]. Its aetiology is multifactorial because of a combination of disease-related immune defects and iatrogenic immunosuppression that affects both humoral and cellular immunity [5, 25].

The prognostic significance of hypogammaglobulinaemia in terms of morbidity, mortality, and overall survival of CLL patients remains controversial. In a study by Rozman et al., the only immunoglobulin class associated with shorter survival in multivariate analysis was IgA [29]. Similarly, in a study by Shvidel et al., IgA was associated with shortened survival, but only in univariate analysis. In multivariate analysis, there was no association between survival and any immunoglobulin class [27]. Andersen et al. described the negative prognostic impact of hypogammaglobulinaemia in all classes on overall survival. However, the largest study on this topic conducted at the Mayo Clinic revealed no such association (although this study tested only IgG) [21]. Morrison et al. focused not on overall survival but on the frequency of infectious complications. In this study, only the levels of IgA were found to be significant [30].

Whereas immunoglobulin substitution therapy is indicated strictly in all CVIDs, in secondary immunodeficiencies, there is a lack of specific markers for identifying patients at higher risk of infections and for determining preemptive IgG replacement therapy. An Italian multicentre prospective cohort study identified specific CVID clinical phenotype characterised by a high pneumonia risk: low IgG and IgA levels at the time of diagnosis, an IgA level < 7 mg/dL, and bronchiectasis [31]. The same increased risk was identified in poor responders to the 23-valent pneumococcal polysaccharide vaccine in the IgA class [32].

Notably, some CLL patients with sufficient immunoglobulin concentrations suffer from recurrent infections, whereas some with hypogammaglobulinaemia do not. Therefore, not only the quantity of immunoglobulins but also the relative levels of IgG subclasses [33, 34] and the ability potential of B cells to form specific immune response are important [35]. Vinsentin et al. examined the best protective cutoff for each immunoglobulin isotype across disease stages, regardless of the therapy provided, with the following results: 7.44 g/L for IgG, 0.79 g/L for IgA, and 0.21 g/L for IgM [22].

As expected, we observed lower levels of all immunoglobulin classes in CLL patients than in healthy controls. However, most patients had IgG levels within the normal range (69.5%), and only 6.7% had IgG levels below 4 g/L. This was expected because a significant proportion of patients had early or indolent disease, and none had been treated for their disease at the time of blood sample collection.

Serious bacterial infections (requiring hospitalization and/or administration of intravenous antibiotics) in our CLL cohort were observed only in 3 of 59 (5%) patients. Thus, we considered analysis of the causal link with immunoglobulin levels statistically meaningless. Although the decreased levels of IgG and anti-PCP IgG did not differ from those in healthy controls, only 12 of 59 (20%) CLL patients had been treated with one (9/59, 15%) or more (3/59, 5%) antibiotic courses due to bacterial infection in the last year. Thus, despite their lower IgG levels and higher anti-PCP levels, these patients had fewer infections. In contrast, low levels of pneumococcal antibodies are associated with severe or multiple infections [23].

Antipneumococcal vaccination is recommended in CLL patients because *S. pneumoniae* is considered a dominant pathogen related to humoral immunodeficiency [11]. The response against polysaccharide antigens may not be sufficient in CLL patients, while conjugate vaccines appear to be more effective [36]. Pasiarski et al. found a statistically significant increase in the titres of specific antipneumococcal IgG following administration of 13-valent pneumococcal conjugate vaccine in CLL patients, although the titres were still much lower in the CLL group than in the control group [28]. Previous studies showed that vaccination can suppress disease progression [37, 38]. B-CLL patients in our study were not vaccinated. Their anti-PCP IgG and anti-PCP IgG2 levels were similar to that of healthy controls, but anti-PCP IgG2 was negatively correlated with disease stage. Although anti-PCP IgG reflects immunoglobulin substitution therapy in CVIDs, anti-PCP IgA levels in this group corresponded with primary immunodeficiency.

There may be a very important vaccination response not only in the IgG class but also in IgA. Our original data revealed lower levels of anti-PCP IgA in nonvaccinated CLL patients than in healthy controls. Total IgA and anti-PCP IgA were negatively correlated with disease stage. The postvaccination response in the IgA class remains unknown. It was not surprising that anti-PCP IgA level in CVID patients was lower than that in healthy controls and the CLL cohort because of more profound defects in humoral immunity.

Re-evaluation of actual antibody production is limited after initiation of substitution immunoglobulin therapy. Specific anti-PCP IgA responses and measured titres of isohaemagglutinins (naturally occurring antibodies mainly of IgM isotypes to polysaccharide blood group antigens) may be an alternative clinically relevant method for assessing T cell-independent antibody production in patients who have already been started on IgG therapy [12].

Natural anti-Gal antibodies represent another helpful marker which can be exploited in assessing antibody production ability. The possibility of determining levels of not only anti-Gal IgG, but also IgA and IgM, allows for evaluation in immunoglobulin-substituted patients. In contrast to specific antipneumococcal antibodies produced in relatively low amount, regarding the specificity, anti-Gal is the most abundant natural antibody in humans. As many as 1% of human B cells can produce anti-Gal, and those along the gastrointestinal tract produce this antibody in response to continuous antigenic stimulation by gastrointestinal bacteria [15]. Mucosal stimulation and production of antibodies in classes IgM as well as IgA are important in defence against infections. These antibodies could serve for assessment of specific humoral immune response in immunodeficient patients.

Little is known regarding anti-Gal production in immunodeficient patients. Subjects with defects in T cell-dependent antibody synthesis may have normal levels of xenoreactive natural antibodies, most of which are specific for Gal alpha 1-3 Gal. Parker et al. described the case of one agammaglobulinaemic patient with undetectable anti-Gal IgM concentration, three patients with severe combined immunodeficiency, and four Wiskott-Aldrich patients with the same findings [39].

We present here the first report of a cohort of 30 CVID patients treated with immunoglobulin substitution therapy and 59 CLL nonsubstituted patients with confirmed decrease in anti-Gal IgA and IgM. The decrease likely depended on the severity of the immunity defect. The level of anti-Gal IgA in the CLL cohort was negatively correlated with disease stage. IgG and anti-Gal IgG levels may be influenced by immunoglobulin substitution therapy. We found no difference between the CVID and CLL groups in the levels of anti-Gal IgM. Our CLL patients were laboratory assessed early after the clinical diagnosis with only low numbers of infections in all clinical stages (Table 1). Therefore, the longer time of follow-up and recruitment of new CLL patients will allow the statistical correlation analysis regarding sinusitis, bronchitis, and pneumonia, thus requiring ATB and the ratio of anti-Gal IgA and IgM to total IgA and IgM, respectively. This is the next point for further investigations.

Moreover, our data suggest that anti-Gal IgA and anti-Gal IgM are highly sensitive markers that can be used to investigate the ability of specific antibody production in patients with primary and secondary immunodeficiency. The results for both isotypes (IgA and IgM) agreed with those for anti-PCP antibodies in immunodeficient patients ([32, 40], Figure 3). Notably, immunization of patients is not required for anti-Gal antibody production, and thus the examination is vaccine-independent and noninvasive.

## 5. Conclusions

Evaluation of antibody production plays a key role in the diagnosis and management of humoral immunodeficiencies. We suggest the use of anti-Gal antibodies in routine testing as an alternative to isohaemagglutinins for the diagnosis of hypogammaglobulinaemia. Significant reductions mainly in anti-Gal IgA, anti-Gal IgM, and anti-PCP IgA may have prognostic importance in immunodeficiency related to CLL. These tests are suitable even in cases of regular immunoglobulin substitution therapy.

## Figures and Tables

**Table 1:** Characteristics of CLL cohort.

Rai stage	0	I	II	III	IV	Total
Patients' number	14	8	14	15	8	59
IgVH mutated/unmutated/NA	10/1/3	3/2/3	3/9/2	1/9/5	3/3/2	20/24/15
TP53 mutated/unmutated/NA	0/6/8	0/5/3	0/12/2	1/8/6	0/5/3	1/36/22
Normal karyotype	3	0	1	5	1	10
13q deletion	7	1	4	3	0	15
12 trisomy	0	1	2	4	3	10
11q deletion	0	2	2	1	2	7
17p deletion	0	0	1	0	0	1
Other karyotype changes	1	3	4	2	1	11
No cytogenetics available	3	1	0	0	1	5
Sinusitis	0	0	2	0	0	2
Bronchitis	2	0	0	0	0	2
Pneumonia	0	0	1	0	1	2
Other bacterial infection requiring ATB therapy	2	1	1	2	2	8
Hospitalisation due to infection	0	0	1	1	1	3

IgVH: immunoglobulin heavy chain variable region gene; TP53: tumour protein 53; NA: data not available. Only prognostically worst karyotype change is recorded; all infections were recorded in the period of one year before blood sample collection.

**Table 2:** Descriptive statistics (all groups).

	CLL (*n* = 59)			CVID (*n* = 30)			Controls (*n* = 67)		
	Median	Q1	Q3	Median	Q1	Q3	Median	Q1	Q3
IgG (g/L)	8.26	5.84	10.75	6.85	5.80	7.88	12.80	10.75	14.95
IgA (g/L)	0.82	0.48	1.69	0.07	0.07	0.14	2.58	1.96	3.12
IgM (g/L)	0.46	0.26	0.81	0.11	0.07	0.20	1.6	0.84	1.42
Anti-Gal IgG (U/mL)	45.88	13.69	80.21	31.60	27.50	39.50	64.01	30.80	144.90
Anti-Gal IgA (U/mL)	5.35	2.93	11.16	0.01	0.01	1.50	25.00	16.12	36.80
Anti-Gal IgM (U/mL)	5.70	1.97	11.31	1.60	0.50	6.68	27.80	14.90	48.68
Anti-PCP IgG (mg/L)	41.10	19.9	68.17	65.05	52.70	79.47	58.30	30.76	108.70
Anti-PCP IgG2 (mg/L)	13.73	7.5	24.49	—	—	—	15.24	7.26	33.10
Anti-PCP IgA (mg/L)	7.58	4.16	17.52	0.63	0.20	2.18	27.00	12.50	55.65

Because the data were not normally distributed, they are shown as the median, Q1 (first quartile), and Q3 (third quartile).

**Table 3:** Descriptive statistics CLL (Rai staging).

Stadium (Rai)	0	I	II	III	IV	Total
IgG (g/L)	10.14	6.80	8.22	7.94	5.80	8.26
	(8.68; 13.18)	(5.36; 8.82)	(7.08; 10.07)	(6.29; 11.15)	(4.15; 7.64)	(5.84; 10.75)
IgA(g/L)	1.75	0.43	1.21	0.73	0.45	0.82
	(1.17; 2.09)	(0.38; 0.53)	(0.76; 1.72)	(0.49; 0.91)	(0.35; 0.83)	(0.48; 1.69)
IgM(g/L)	0.79	0.29	0.43	0.30	0.24	0.46
	(0.57; 0.83)	(0.21; 0.45)	(0.33; 0.65)	(0.20; 0.62)	(0.12; 0.47)	(0.26; 0.81)
Anti-Gal IgG (U/L)	65.32	35.80	29.70	49.78	10.42	45.88
	(18.73; 132.10)	(2.73; 50.51)	(0.78; 57.09)	(4.38; 85.90)	(1.78; 86.15)	(13.69; 80.21)
Anti-Gal IgA (U/mL)	11.80	2.37	5.9	4.70	2.84	5.35
	(8.23; 16.39)	(1.56; 6.31)	(3.05; 8.33)	(3.38; 9.19)	(1.07; 5.19)	(2.93; 11.16)
Anti-Gal IgM (U/mL)	10.22	9.41	3.74	6.87	1.89	5.7
	(6.19; 20.05)	(2.42; 13.97)	(1.75; 7.57)	(1.59; 11.96)	(1.25; 3.35)	(1.97; 11.31)
Anti-PCP IgG (mg/L)	62.66	45.72	30.4	42.58	20.86	41.10
	(38.18; 89.64)	(36.57; 57.67)	(15.69; 56.06)	(15.42; 73.97)	(17.32; 37.50)	(19.09; 68.17)
Anti-PCP IgG2 (mg/L)	24.28	19.10	10.86	10.19	7.14	13.73
	(13.96; 29.86)	(14.80; 23.43)	(5.32; 18.38)	(4.48; 21.35)	(6.60; 11.43)	(7.05; 24.49)
Anti-PCP IgA (mg/L)	15.64	6.41	10.57	6.97	3.74	7.58
	(9.62; 22.58)	(4.20; 10.30)	(5.65; 24.78)	(3.79; 9.37)	(3.23; 4.67)	(4.16; 17.52)

Because the data were not normally distributed, they are shown as the median, Q1 (first quartile), and Q3 (third quartile).

**Table 4:** Comparison between groups.

	CLL × controls	CLL × CVID	CVID × controls
IgG	<0.0001	1	<0.0001
IgA	<0.0001	<0.0001	<0.0001
IgM	<0.0001	0.0001	<0.0001
Anti-Gal IgG	0.3303	1	0.0180
Anti-Gal IgA	<0.0001	0.0002	<0.0001
Anti-Gal IgM	<0.0001	0.3504	<0.0001
Anti-PCP IgG	ND	ND	ND
Anti-PCP IgG2	ND	ND	ND
Anti-PCP IgA	<0.0001	<0.0001	<0.0001

ND: Wilcoxon two-sample rank-sum test was not performed because the *p* value was more than 0.05 in Kruskal-Wallis test.
